# Supplementary material for: Tumour-derived PGD2 and NKp30-B7H6 engagement drives an immunosuppressive ILC2-MDSC axis
Source: Nat Commun. 2017 Sep 19;8:593. doi: 10.1038/s41467-017-00678-2 (PMC5605498; doi:10.1038/s41467-017-00678-2)
Supplement: Supplementary file 2 — Supplementary Information [file 41467_2017_678_MOESM2_ESM.pdf]

Description of Supplementary Files

File name: Supplementary Information
Description: Supplementary figures

File name: Peer Review File

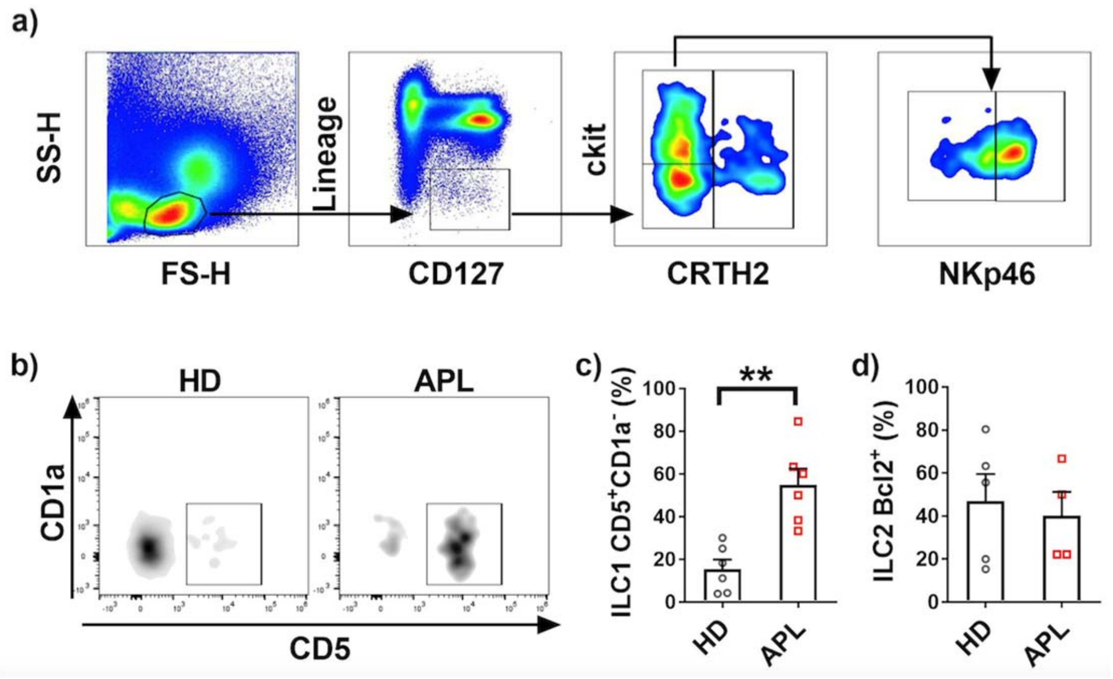

Supplementary Figure 1. Identification of ILCs and ILC precursors. a) Mononuclear cells were stained for Lineage markers, CD127, CRTH2, cKit and NKp46, then gated on Lin$^-$CD127$^+$ cells, as depicted. Among Lin$^-$CD127$^+$ cells, ILC subsets were defined as follow, ILC1: Lin$^-$CD127$^+$CRTH2$^-$cKit$^-$; ILC2: Lin$^-$CD127$^+$CRTH2$^+$cKit$^{+/-}$; ILC3 NCR$^+$: LinCD127$^+$CRTH2$^-$cKit$^+$NKp46$^+$; ILC3 NCR$^-$: Lin$^-$CD127$^+$CRTH2$^-$cKit$^+$NKp46$^-$. **b)** Representative examples of flow cytometry analysis of precursors CD5$^+$CD1a$^-$ of ILCs in the bone marrow of healthy donors (HD) and APL patients (APL). **c)** Frequencies of CD5$^+$CD1a$^-$ ILC precursors within the ILC1 subset in HD (n=6) and APL (n=6). **d)** Frequency of Bcl2 expressing ILC2 in HD and APL PB (n=4). Error bars are s.e.m. Statistical analysis was performed using Mann-Whitney test (c-d).

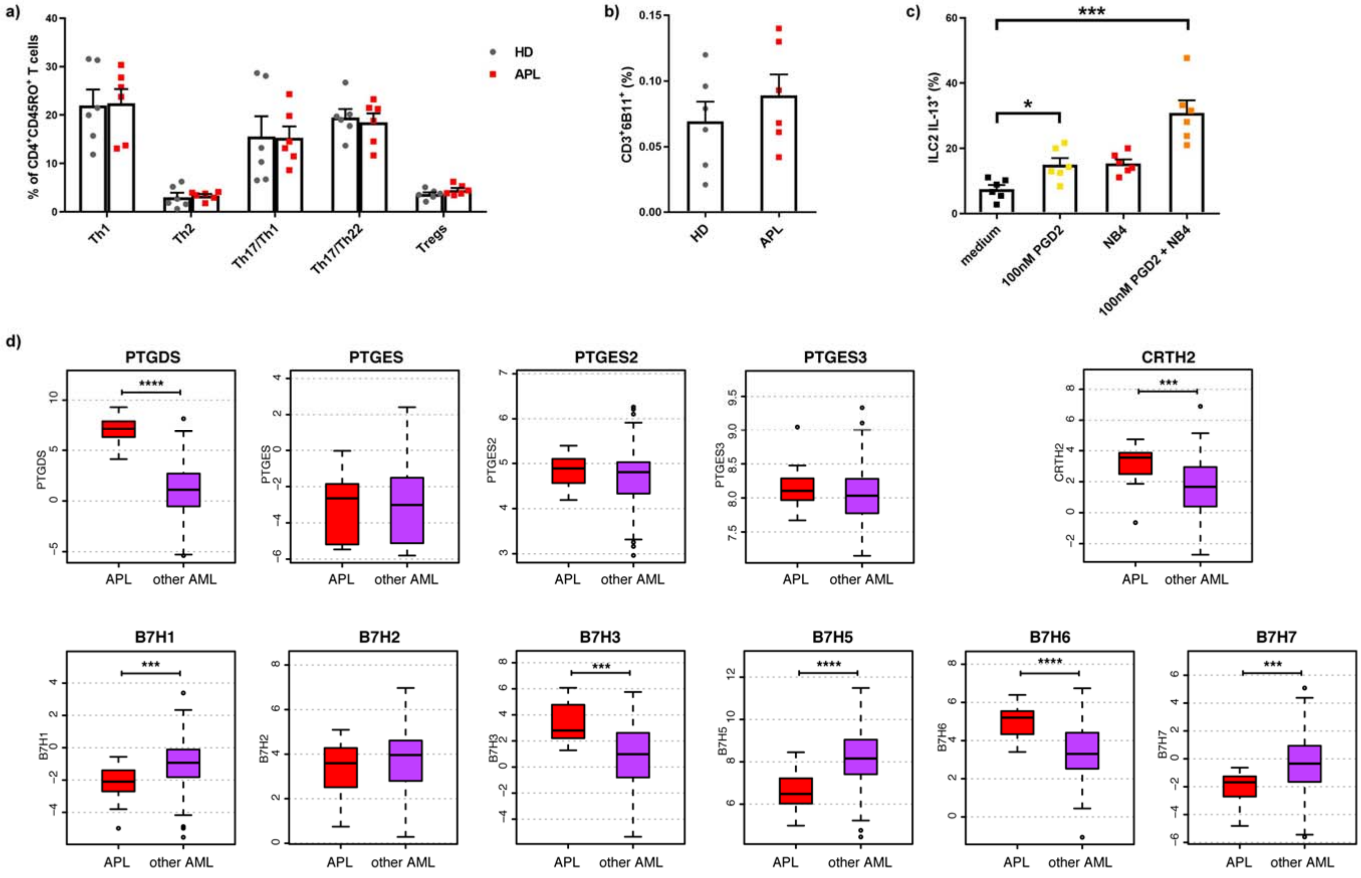

Supplementary Figure 2. Immune profiling in APL patients. a) Relative frequencies of T-helper subsets and Regulatory T cells (Tregs) among memory CD45RO$^+$CD3$^+$CD4$^+$ T cells in healthy donors (HD, n=6) and APL patients (APL, n=6). CD4$^+$ T cell subsets are defined as: Th1: CXCR3$^+$CCR6$^-$; Th2: CXCR3$^-$CRTH2$^+$; Th1/Th17: CXCR3$^+$CCR6$^+$; Th17/Th22: CXCR3$^-$CCR6$^+$CCR10$^+$; Tregs: CD25$^+$CD127$^-$. **b)** Relative frequencies of NKT cells among lymphocytes in healthy donors (HD, n=6) and APL patients (n=6). **c)** Frequencies of IL-13 producing ILC2s upon 24 hours co-culture with PGD2, the APL cell line NB40 or both (6 independent experiments). Co-cultures were performed using enriched ILCs from peripheral blood, and IL-13 secretion was monitored on Lin$^-$ CD127$^+$CRTH2$^+$ cells. **d)** mRNA expression levels for prostaglandin synthases (PTGDS, PTGES, PTGES2, PTGES3), CRTH2 and B7H molecules retrieved from The Cancer Genome Atlas (TCGA) for APL patients (APL, n=16) as compared to patients with all the other AML subtypes (other AML, n=155). B7H4 mRNA expression is not represented, since it was undetectable. Boxplots of selected genes' expression indicate the median, the first and third quartiles as well as outliers. Error bars are s.e.m. Statistical analysis was performed using Mann-Whitney test (a, b) and Kruskal-Wallis test (c).

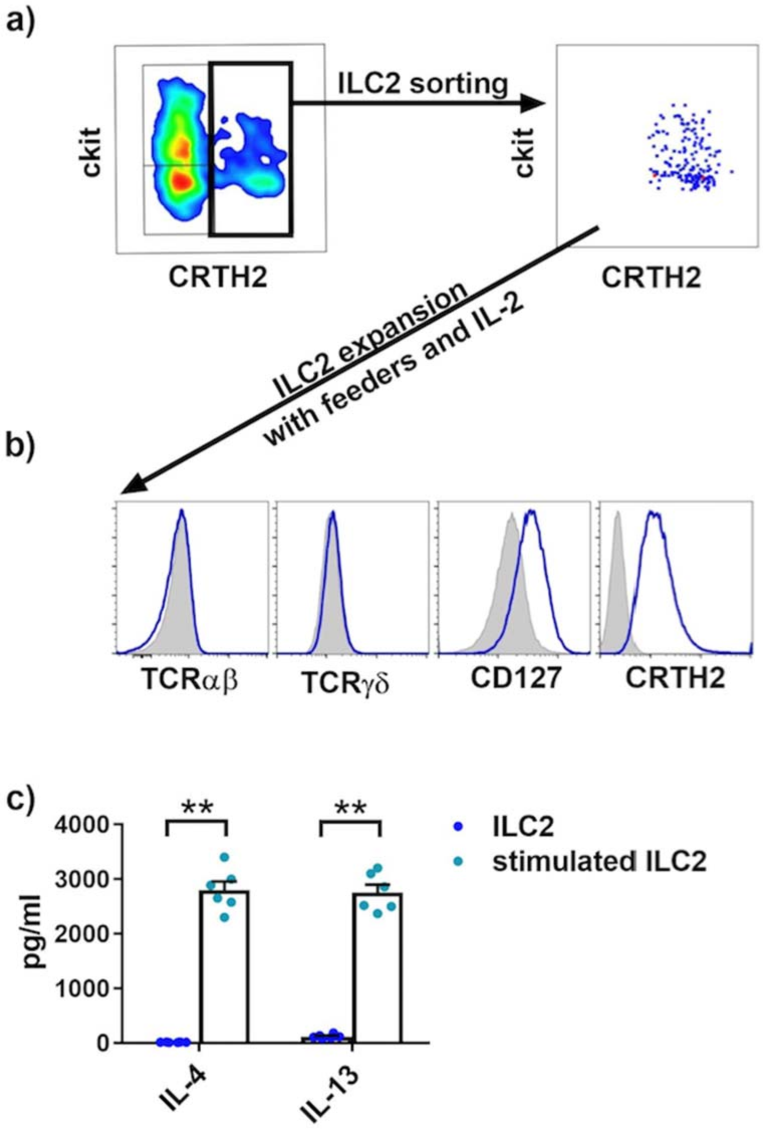

Supplementary Figure 3. Characterization of ILC2 cell lines. **a)** Gating strategy used for the sorting of highly-pure ILC2 cells for the generation of short-term expanded ILC2 lines. ILC2 cells were selected within Lin^- $CD127^+$ living lymphocytes and sorted based on CRTH2 expression. After purity check ILC2 were short-term expanded *in vitro*. **b)** Representative examples of flow cytometry analysis of an ILC2 cell line. Isotype control in grey. **c)** Quantification of prototypic ILC2 cytokines (i.e. IL-4 and IL-13) in the supernatant of ILC2 line in steady state condition (ILC2) or upon PMA-Ionomycin stimulation (stimulated ILC2). Error bars are s.e.m. Statistical analysis was performed using Mann-Whitney test (c).

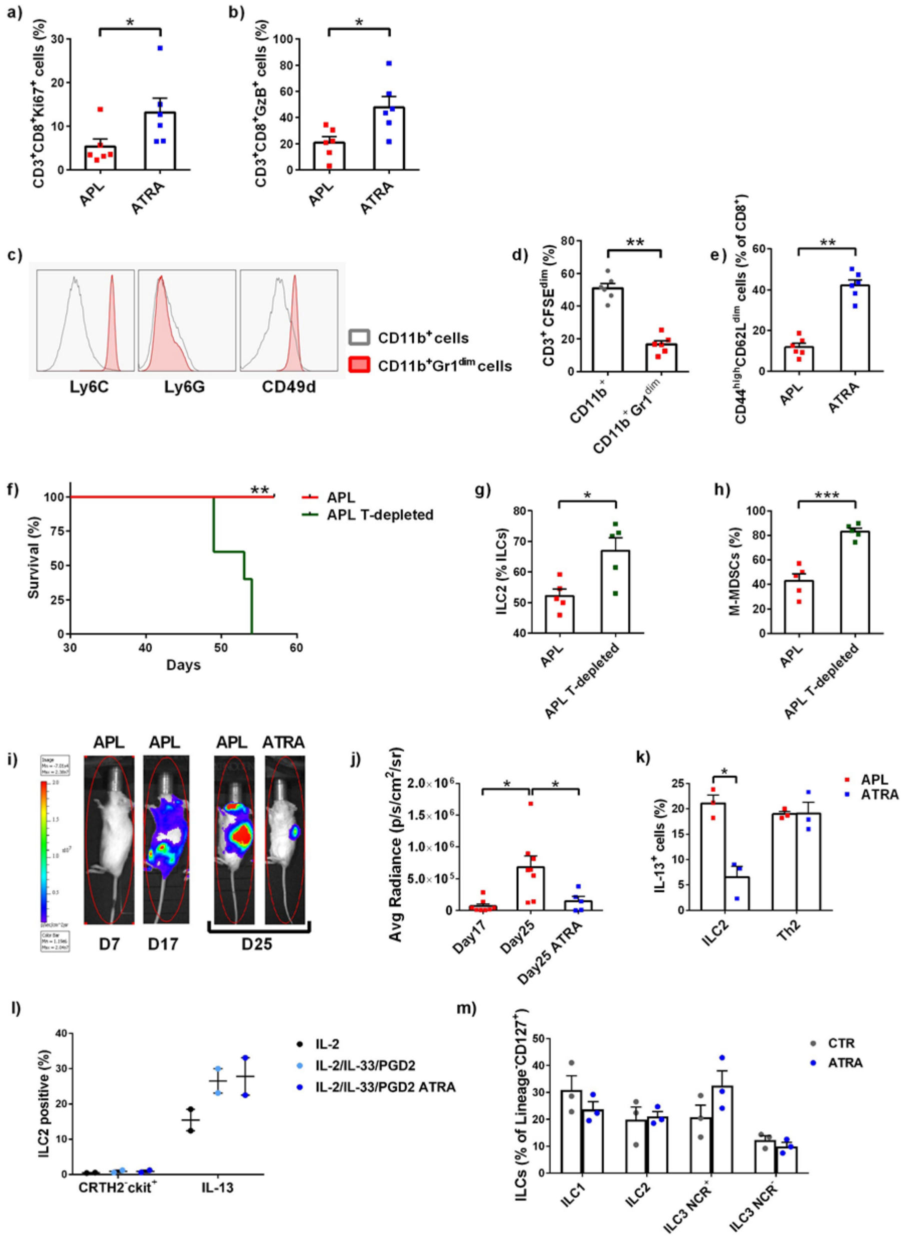

Supplementary Figure 4. ***Bona fide*** **M-MDSC inhibit the anti-tumor response mediated by the T cell compartment in APL.** Frequencies of **a)** Ki67 and **b)** Granzyme B positive cells among $CD3^{+}CD8^{+}$ cells in APL patients at diagnosis (APL) or in remission (APL Rem; n=6). **c)** Representative examples of phenotypic characterization of murine M-MDSC by evaluation of the expression of Ly6C, Ly6G and CD49d (n=3), as previously described[51,52]. **d)** Frequencies of proliferating purified $CD3^{+}$ T cell upon co-culture with purified murine M-MDSC (n=6). **e)** Frequency of activated $CD44^{high}$ $CD62L^{dim}$ $CD3^{+}CD8^{+}$ T cells in mice injected with APL blasts, untreated (APL) or after ATRA treatment (ATRA; n=6). **f)** Survival curves, **g)** ILC2 frequencies and **h)** M-MDSC frequencies of mice injected with APL blasts, untreated (APL) or upon T-cell depletion (APL T-depleted; n=5). **i)** Representative examples and **j)** cumulative data of luminescence measurement for quantification of leukemic engraftment in untreated APL HIS mice (APL, day 17 n=8, day 25 n=8) and mice treated with ATRA (ATRA, Day 25 n=5). **k)** Frequencies of IL-13^{+} ILC2 and Th2 cells upon APL cell line stimulation. Cells were isolated from untreated APL HIS mice (APL) or APL HIS mice after ATRA treatment (ATRA; n=3). **l)** Phenotype and frequency of IL-13^{+} producing cells among highly-pure human ILC2 lines cultured overnight in medium supplemented with IL-2, IL-33, PGD2 and ATRA, as indicated. Conversion to ILC3 was evaluated by the quantification of $CRTH2^{-}cKit^{+}$ cells, IL-13 production was assessed by intracellular cytokine staining (n=2)**, m)** Frequency of ILC subsets in HIS mice untreated (CTR) and after ATRA administration (ATRA) (n=3). Error bars are s.e.m. Statistical analysis was performed using Mann-Whitney test (a-h, k), Log-Rank test (Mantel-Cox) (f) and Kruskal-Wallis test (j).

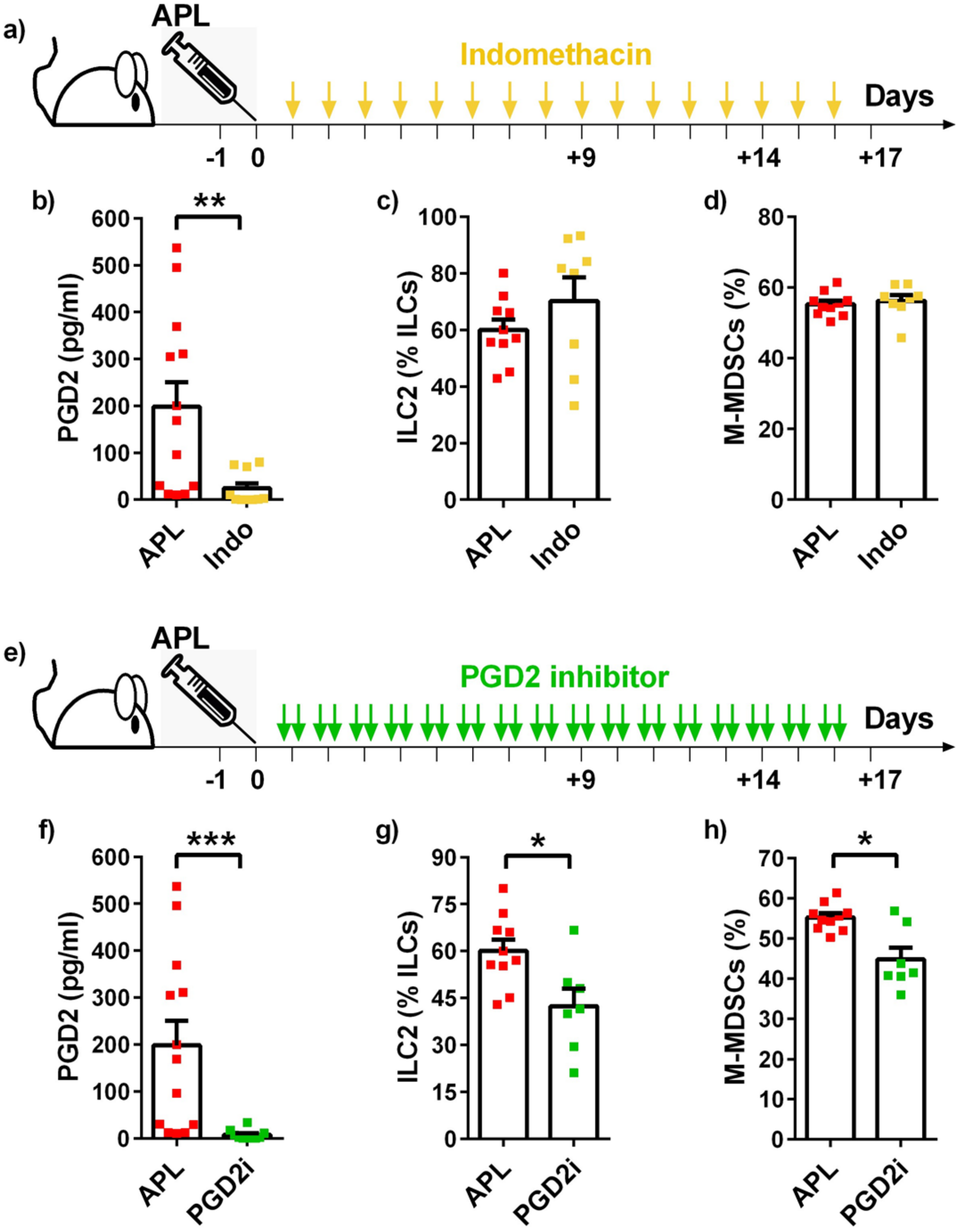

Supplementary Figure 5. Specific inhibition of PGD2 partially reverses the PGD2-ILC2-IL-13-M-MDSC immunosuppressive axis in an APL mouse model. a) Schematic representation of the schedule of Indomethacin treatment in established APL in FVB/NJ mice (2 independent experiments). **b)** Comparison of serum concentrations of PGD2 (APL n=13, Indo n=10), **c)** ILC2 frequencies (APL n=10, Indo n=8) and **d)** M-MDSC frequencies in APL mice (APL, day 13-17) or APL mice after Indomethacin treatment (Indo, day 14-17) (APL n=10, Indo n=8). **e)** Schematic representation of the schedule of PGD2 inhibitor treatment (PGD2i, TM30089) in FVB/NJ injected with APL blasts (2 independent experiments). **f)** Comparison of serum concentrations of PGD2 (APL n=13, PGD2i n=10), **g)** ILC2 frequencies (APL n=10, PGD2i n=7) and **h)** M-MDSC frequencies in peripheral blood of APL mice (APL, day 13-17) or APL mice after TM30089 treatment (PGD2i, day 15-17) (APL n=10, PGD2i n=7). Error bars are s.e.m. Statistical analysis was performed using Mann-Whitney test.

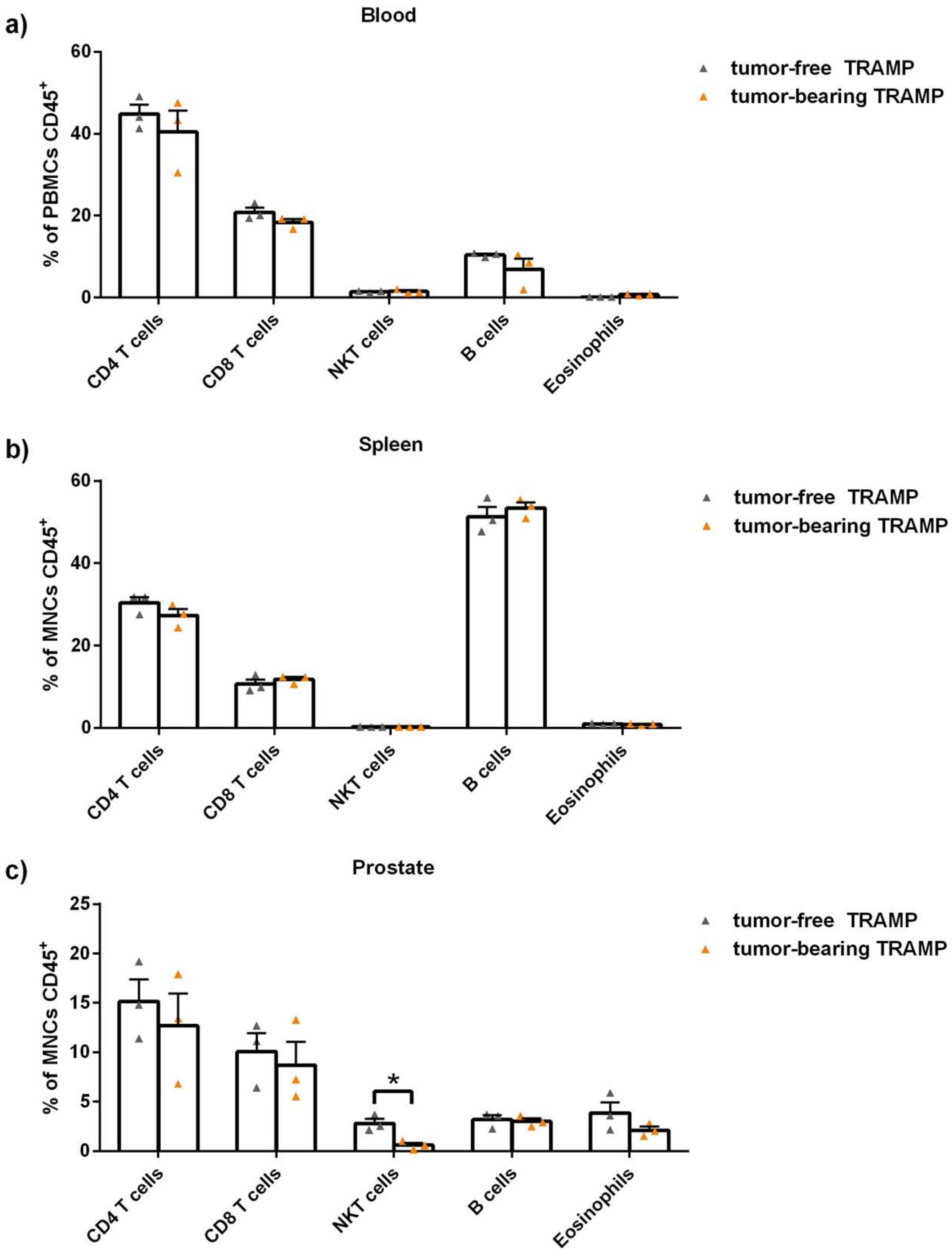

Supplementary Figure 6. Quantification of different immune cell populations in TRAMP mice. $CD4^+$ T cells, $CD8^+$ T cells, NKT cells, B cells and eosinophils were quantified in the blood **a),** spleen **b)** and prostate **c)** of tumor- free or tumor-bearing TRAMP mice (n=3). Error bars are s.e.m. Statistical analysis was performed using Mann-Whitney test.
